# Genomic indicators of gene function: a systematic assessment of the human genome

**DOI:** 10.1093/g3journal/jkag215

**Published:** 2026-08-22

**Authors:** Helena B Cooper, Karla E Rojas López, Daniela Schiavinato, Michael A Black, Paul P Gardner

**Affiliations:** Department of Biochemistry, Faculty of Biomedical Sciences, University of Otago, Dunedin 9016, New Zealand; Department of Biochemistry, Faculty of Biomedical Sciences, University of Otago, Dunedin 9016, New Zealand; Department of Biochemistry, Faculty of Biomedical Sciences, University of Otago, Dunedin 9016, New Zealand; Department of Biochemistry, Faculty of Biomedical Sciences, University of Otago, Dunedin 9016, New Zealand; Department of Biochemistry, Faculty of Biomedical Sciences, University of Otago, Dunedin 9016, New Zealand

**Keywords:** genome annotation, functional genomics, human genome, evolutionary conservation, non-coding RNA, comparative genomics, transcriptomics, RNA biology

## Abstract

Proteins and non-coding RNAs are functional products of the genome that are central for crucial cellular processes. With recent technological advances, researchers can sequence genomes in the thousands and probe numerous genomic activities of many species and conditions. Such studies have identified thousands of potential proteins, RNAs, and associated activities. However, there are conflicting interpretations of the results and therefore which regions of the genome are “functional.” Here we investigate the relative strengths of associations between coding and non-coding gene functionality and genomic features, by comparing reliably annotated functional genes to nongenic regions of the genome. We find that the strongest and most consistent associations between functional genes and genomic features are transcriptional activity and evolutionary conservation. We also evaluated sequence-based statistics, genomic repeats, and epigenetic and population variation data. Other features strongly associated with function include histone marks, chromatin accessibility, genomic copy number, and sequence alignment statistics such as coding potential and covariation. We also identify potential issues with SNP annotations in short non-coding RNAs, as some highly conserved ncRNAs have significantly higher than expected SNP densities. Our results demonstrate the importance of evolutionary conservation and transcription activity for indicating protein-coding and non-coding gene function. Both should be taken into consideration when differentiating between functional sequences and biological or experimental noise.

## Introduction

A substantial portion of the human genome—particularly intergenic and intronic regions—is composed of repeat-derived, non-protein-coding sequences that remain poorly understood. These regions often evolve at neutral rates and appear to contribute little to organismal fitness. As a result, they have historically been labeled as “junk DNA” (Doolittle 2013; Germain et al. 2014; Rands et al. 2014; Doolittle and Brunet 2017). The term serves not only as a descriptor but also as a null hypothesis in genomics, where function must be demonstrated, not assumed (Koonin 2016).

This perspective stands in contrast to claims from projects such as ENCODE, which argue that widespread biochemical activity, particularly transcription, is indicative of function (Dinger et al. 2009; Mattick et al. 2010; ENCODE Project Consortium 2012; ENCODE Project Consortium et al. 2020). However, critics argue that biochemical activity alone is insufficient, as it can arise from biological noise, leaky transcription, or experimental artifacts. According to the “selected effect” definition of function, activity must also be associated with fitness effects or evolutionary constraint (Eddy 2012; Doolittle et al. 2014; Germain et al. 2014; Palazzo and Gregory 2014; Doolittle and Brunet 2017).

Indeed, numerous studies (Haerty and Ponting 2014; Christmas et al. 2023) have shown that much of the genome is evolutionarily indistinguishable from neutral sites such as ancestral repeats or 4-fold degenerate sites, providing empirical support for this more conservative view. These findings reinforce the need to combine evolutionary and biochemical evidence to confidently define functional regions.

Defining functional elements in the human genome remains challenging (Kellis et al. 2014; Tsai et al. 2017). Many observed biochemical activities, such as transcription, may simply reflect biological noise or experimental artifacts (Raser and O'Shea 2005; Eddy 2012; Doolittle 2013; Graur 2017). Random transcription can arise from nonspecific DNA-binding proteins, indiscriminate promoters, or transcribed transposons, and random sequences are increasingly shown to yield bioactive RNAs or peptides (Dube et al. 1993; Raser and O'Shea 2005; Struhl 2007; Fajardo 2021; Frumkin and Laub 2023; Hlouchova 2023; Luthra et al. 2024). Ongoing efforts are working to establish baseline biochemical activity in the absence of selection (Eddy 2024).

Biochemical activity alone does not resolve the C-value paradox, where genome size varies widely without corresponding changes in perceived phenotypic complexity (Eddy 2012). For example, closely related vizcacha rats differ 2-fold in genome size yet are phenotypically very similar (Evans et al. 2017). The genome size disparities are largely explained by the accumulation of selfish, repeat-derived sequences, particularly transposable elements (TEs), which evolve predominantly under neutral drift (Arkhipova 2018; Osmanski et al. 2023). While some TEs have been co-opted for host function, generalizing from a few well-characterized cases to widespread functionality risks confirmation bias and circular reasoning (Lowe and Haussler 2012; Doolittle and Brunet 2017; Bourque et al. 2018; Hayward and Gilbert 2022).

We hypothesize that functional coding and non-coding genes can be distinguished from the genomic background, which is largely composed of decayed or inactive repeat elements (Bourque et al. 2018; Frankish et al. 2019; Klapproth et al. 2021). To test this, we systematically evaluate a broad set of genomic features previously linked to functionality, using consistent and controlled statistical comparisons. Note that long non-coding RNA (lncRNAs) are highly heterogeneous; therefore, these genome-wide comparisons should be interpreted as class-level summaries, not as a substitute for evaluations of individual loci. Our analysis highlights the most informative signals for defining function and helps clarify the criteria needed to robustly distinguish genic regions from nonfunctional DNA.

## Materials and methods

We next describe the selection of positive and negative control sequences, the genomic features included, and the statistical methods used. For each gene class, we analyzed 1,000 positive and up to 10,000 negative genomic regions. The 10-to-1 negative-to-positive ratio was chosen to reflect the genomic excess of unconserved over conserved sequence inferred from evolutionary analyses (Christmas et al. 2023).

### Retrieval of functional genes

We designated sequences as “functional” based on annotations from the HUGO Gene Nomenclature Committee (HGNC) database, which links each gene to documented evidence of function (Braschi et al. 2019). Because named genes undergo multiple rounds of expert curation, we considered them more likely to be functional.

We retrieved protein-coding sequence IDs from HGNC and filtered out withdrawn entries or those with multiple RefSeq IDs (Braschi et al. 2019). Using these RefSeq IDs, we extracted chromosome coordinates and corresponding sequences from the GRCh38.p14 genome (O’Leary et al. 2016).

To include functional non-coding RNAs, we sourced short non-coding RNAs (sncRNAs) and multi-exonic lncRNAs from RNAcentral (release 22.0), including only those with assigned HGNC ID. We downsampled precursor miRNAs, which are overrepresented in RNAcentral, to balance the representation of different sncRNAs types (Freyhult et al. 2005; The RNAcentral Consortium 2019). Our sncRNA dataset includes small nucleolar RNAs (snoRNAs) (*n* = 393, 39.3%), transfer RNAs (tRNAs) (*n* = 268, 26.8%), small nuclear RNAs (snRNAs) (*n* = 7, 0.7%), microRNAs (miRNAs) (*n* = 302, 30.2%), and other less represented groups such as vault (*n* = 4, 0.4%), sca (*n* = 13, 1.3%), and snaR RNAs (*n* = 13, 1.3%).

We excluded chromosome Y and mitochondrial DNA due to the former's excess low-complexity sequence, and the latter’s different selection pressures (Quintana-Murci and Fellous 2001). We also excluded ribosomal RNAs because of their large sequence length, unusual expression and sequence conservation patterns, and uncertain placement in the reference human genome (Agrawal and Ganley 2018).

From each functional category, we randomly selected up to 1,000 genes. For protein-coding genes, we created 2 datasets: one for exon 2 and another for exon 3, focusing on internal exons and avoiding untranslated regions and promoters, which are subject to different evolutionary constraints. For lncRNA genes, we generated separate datasets for exon 1 and exon 2, due to the limited number of multi-exonic annotations.

To minimize length-dependent effects and ensure computational feasibility, given that some of the algorithms scale poorly with sequence length, we filtered sequences by length. We defined the upper and lower limits as the 10th and 90th percentiles of the length distribution of each RNA class: protein-coding RNA (61 to 271 nucleotides), lncRNA (74 to 1,500 nucleotides), and sncRNA (71 to 142 nucleotides). This approach preserved statistical robustness while maintaining manageable input sizes for downstream analyses.

### Negative control dataset

To generate the negative control datasets, we sampled up to 10 negative control sequences per functional sequence from the GRCh38.p14 human genome (O’Leary et al. 2016). We filtered these sequences to exclude any overlaps with annotated genes. Using Bedtools 2.29.0, we removed controls that intersect with RNAcentral and GENCODE annotations (Harrow et al. 2012; Quinlan 2014; Haeussler et al. 2019). We selected these databases for their extensive annotation coverage, significant overlap with other resources (GENCODE v45), and the high number of unique entries provided by RNAcentral (Supplementary Fig. 1).

We chose control coordinates from regions located approximately 1, 10, 100, 1,000, and 5,000 kb upstream and downstream of each functional sequence. These distances allowed us to sample broadly across “nongenic” regions and assess the influence of local genomic signatures (Chor et al. 2009; Costantini et al. 2009; Elhaik and Graur 2014) (Supplementary Fig. 2).

To control for sequence length bias, we matched each negative control sequence to the length to its corresponding functional sequence. We excluded sequences with more than 5% ambiguity (ie “N”).

We deliberately set the functional-to-control ratio at approximately 1:10, reflecting recent estimates of the proportion of the genome under functional constraint and negative selection (Christmas et al. 2023). This ratio aligns with our broader goal of applying these features to large genomic regions—or even genome-wide—where most sequences are expected to be nonfunctional.

### Feature inclusion criteria

Genomic features were included if they met the following criteria: (i) prior evidence of association with gene function; (ii) applicable to genome-wide analyses including the negative controls; (iii) informative without redundancy; (iv) minimal bias toward known genes (eg not homology-based); (v) available for GRCh38; and (vi) accessible, installable software where required.

Starting from an initial pool of 98 features, we applied these criteria and excluded 72 that failed to meet 1 or more requirements. We retained 30 features for downstream analysis (Supplementary Tables 1 and 2) and included a baseline pseudorandom number (“Random”).

### Transcriptome expression features

Transcriptional activity was estimated using ENCODE RNA-seq datasets comprising 71 human tissues and 140 primary-cell samples (ENCODE Project Consortium 2012). Libraries were rRNA-depleted; total RNA-seq captured transcripts (≥200 bp), and small RNA-seq targeted transcripts (<200 bp). Dataset accession IDs are provided in the project repository (Supplementary Table 3). Expression levels were quantified as RPKM (reads per kilobase of transcript per million mapped reads) using SAMtools v1.10 (Li et al. 2009), and the maximum RPKM across all datasets was recorded for each sequence. The maximum RPKM was chosen to capture tissue- or condition-specific expression, which may be obscured by averaging across diverse samples, although this approach may be sensitive to outliers and technical variability.

### Sequence conservation features

We extracted phyloP scores to analyze sequence conservation from the UCSC hg38 phyloP 100-way and phyloP 241-way bigWig files, which represents multiple alignments of 100 vertebrate species and 241 placental mammals, respectively. To obtain these scores, we used the bigWigSummary command-tool from the UCSC Genome Browser's “kent” bioinformatic utilities (v385) (Haeussler et al. 2019). We also retrieved GERP (Genomic Evolutionary Rate Profiling) scores for each region using Bedtools 2.29.0, based on the release of 111 Ensembl multiple genome alignments that included 91 eutherian mammals and 63 amniote vertebrates (Cooper et al. 2005; Quinlan 2014; Yates et al. 2020). For each region and method, we used the maximum score as the summary statistic, as this consistently produced the strongest effects in our preliminary evaluation.

### Epigenetic signature features

To assess the contribution of epigenetic features to gene functionality, we obtained processed ENCODE3 datasets (narrowPeak and bedMethyl formats) spanning ∼110 tissues, 91 cell lines, 33 in vitro differentiated cells, and 76 primary-cell types (Davis et al. 2018; ENCODE Project Consortium et al. 2020) (Supplementary Table 3). From an initial set of 32 histone modifications, we selected H3K79me1, H3K79me2, and H3K9ac using a 2-step feature selection procedure: (i) Kolmogorov–Smirnov (KS) tests to identify features that best distinguish genic and control regions, followed by (ii) Spearman correlation analysis to retain informative, minimally redundant marks (Supplementary Fig. 3). Chromatin accessibility and CpG methylation were included as additional epigenetic features.

We use an approach that is consistent with the transcriptome analysis above. For each feature, we calculated the maximum signal across all samples and scaled by the degree of overlap between control sequence annotations and the corresponding histone marks or chromatin accessibility regions, ie


$$\mathrm{EpiScor}e_{i}=\;\frac{\mathrm{ma}x_{j}\;(\mathrm{ma}x_{k}(v_{k}*\;\mathrm{overla}p_{k}))}{L_{k}}\;$$

For each control sequence ($\mathrm{EpiScor}e_{i}$), signal values (*v_k_*) are obtained from narrowPeak ENCODE files, which give enrichment values using local background normalization (Feng et al. 2012). The overlap*_k_* value is the overlap length between the control sequence *i* and histone mark or chromatin accessibility region *k*. *L_k_* is the length of control sequence *i*. The max signal is chosen across all *j* tissues and cell lines.

For methylome measures, we used a different approach to account for saturated sites, which becomes inevitable given enough samples. Instead, we calculated an average proportion of methylations per CpG site as follows:


$$\mathrm{Met}h_{i}=\;\frac{\sum_{\;j=1,k=1}^{s_{j},n_{k}}\;(\;p_{\;jk})}{n_{k}*\;s_{j}}$$

where $\mathrm{Met}h_{i}$ is the average proportion of CpG methylations per sample per CpG site for sequence *i*. The proportions *p_jk_* are the proportion of methylated sites identified at site *k* in sample *j* (*s_j_*). The number *n_k_* is the number of CpG sites within each control sequence.

### Protein and RNA-specific features

We analyzed protein and RNA-specific features using multiple sequence alignments (MSAs), coding potential metrics, RNA structural properties, and RNA:RNA interaction profiles. To obtain MSAs, we extracted alignments from the Zoonomia 241 Placental Mammals dataset (Zoonomia Consortium 2020) using the bigBedToBed command-line tool from UCSC Genome Browser's “kent” bioinformatic utilities (v385) (Lorenz et al. 2011; Haeussler et al. 2019; Zoonomia Consortium 2020). We filtered out partial alignments that covered less than 50% of the *Homo sapiens* reference sequence.

We assessed coding potential using CPC2 beta and RNAcode (ViennaRNA 2.4.14), applying either Fickett scores derived from individual sequences, or RNAcode scores calculated from the corresponding MSAs (Lorenz et al. 2011; Washietl et al. 2011; Kang et al. 2017).

To evaluate RNA structure, we used R-scape 1.4.0 to calculate the maximum covariance score (*G*-test) for each region based on the MSA (Rivas et al. 2017). We also applied RNAfold from ViennaRNA 2.4.14 to compute the minimum free energy (MFE) value for each sequence (Lorenz et al. 2011; Bhandari et al. 2021).

To explore RNA–RNA interactions and potential avoidance mechanisms, we used RNAup 2.3.3 to calculate average interaction free energies between each sequence and a curated database of 34 abundant human ncRNA sequences (Supplementary Table 4) from RNAcentral v15, which are excluded from the functional dataset (The RNAcentral Consortium 2019). These ncRNAs are known to interact with a variety of RNAs (Marvin et al. 2011; Mann et al. 2017; Driedonks and Nolte-’t Hoen 2018; Yan et al. 2019).

Canonical splice-site propensity was estimated with MaxEntScan, which scores matches to 5′ and 3′ splice motifs (Yeo and Burge 2004). Each region, including 50 nucleotides of flanking sequence on either side, was scored with both models, and the maximum 5′ and 3′ scores are summed and recorded for that region.

### Genomic repeat-associated features

We estimated the genomic copy number of each sequence by aligning it against the GRCh38.p14 human genome using MMseqs2 v15.6f452 (Hauser et al. 2016). We included all sequence matches with an *E*-value smaller than 0.001. To calculate the “repeat-free” feature, we used nonredundant hits of repetitive DNA elements in the human genome obtained from Dfam v3.8. These annotations include TEs (LINEs, SINEs, LTRs, DNA transposons, other interspersed repeats), satellite repeats, pseudogenes, and conserved repeats, identified via profile hidden Markov models.

We identify the closest repetitive element up- and downstream of the sequence using Bedtools 2.29.0 (Quinlan 2014; Hubley et al. 2016). We excluded repeats that overlapped with the control regions, as many small ncRNAs are classified as repeats (eg tRNAs, spliceosomal RNAs). For each sequence, we recorded the sum of the distances to the nearest up- and downstream repeats, following a strategy similar to the historic “repeat-free” approach (Simons et al. 2006).

### Population variation features

To assess population variation, we calculated SNP density and average minor allele frequency (MAF) for each genomic region. Variant data were obtained from gnomAD v4 VCF files using Tabix v1.10 (Li 2011; The 1000 Genomes Project Consortium 2015; Karczewski et al. 2020). SNP density is defined as the number of SNPs per base pair, and average MAF is the mean MAF across all SNPs within each sequence.

### Intrinsic sequence features

We calculated G + C content by determining the percentage of guanines and cytosines in each sequence and mononucleotide frequencies as the percentage of each nucleotide (A, C, G, T). To assess low complexity regions (LCRs), we used DUST 1.0.0 (Morgulis et al. 2006) to compute low complexity density across the dataset.

For each dinucleotide, we calculated a log-odds (bitscore) which quantifies observed dinucleotide frequencies relative to expected frequency based upon mononucleotides (eg CpG frequency corrected for C and G frequencies):


$$\begin{array}{rl} \mathrm{bits}= & \mathrm{lo}g_{2}\left(\frac{\;f(xy)}{\;f(x)\;\times \;f(y)}\right)\\ \approx & \mathrm{lo}g_{2}(\;f(xy)+p)\;-\;\mathrm{lo}g_{2}\left(\;f(x)+\sqrt{p}\right)\;-\;\mathrm{lo}g_{2}\left(\;f(y)+\sqrt{p}\right) \end{array}\;$$

where


$$f(xy)=\frac{\mathrm{count}(xy)}{\mathrm{length}(\mathrm{seq})-\mathrm{length}(k\mathrm{mer})+1},\;f(x)=\frac{\mathrm{count}(x)}{\mathrm{length}(\mathrm{seq})},\;f(y)=\frac{\mathrm{count}(y)}{\mathrm{length}(\mathrm{seq})}.$$

0 < *f*(*xy*) ≤ 1 is the frequency of dinucleotide *xy*, and 0 < *f*(*x*) ≤ 1 and 0 < *f*(*y*) ≤ 1 are the frequencies of nucleotides *x* and *y*, respectively. A small pseudocount (*P* = 0.01) was added to avoid zero values, particularly in short sequences, and to ensure stable log-ratio estimates.

### Excluded features

Features that did not meet these inclusion criteria were excluded. Most were removed because they were uninformative in a small independent test set, redundant with stronger features, not genome-wide, or unavailable for GRCh38. Supplementary Table 2 lists all excluded features and the reasons for exclusion. These included, among others, GTEx expression, PhastCons scores, additional histone marks, PhyloCSF scores, some covariation summaries, INDEL-free regions, Tajima's *D*, and transition:transversion ratios.

### Random number

To establish a null baseline, each sequence was assigned a random integer (0 to 32,767) generated using the Bash $RANDOM function, which produces approximately uniformly distributed pseudorandom values. This feature was included as a control to identify signals no stronger than random expectation (Fig. 1).

**Fig. 1.**
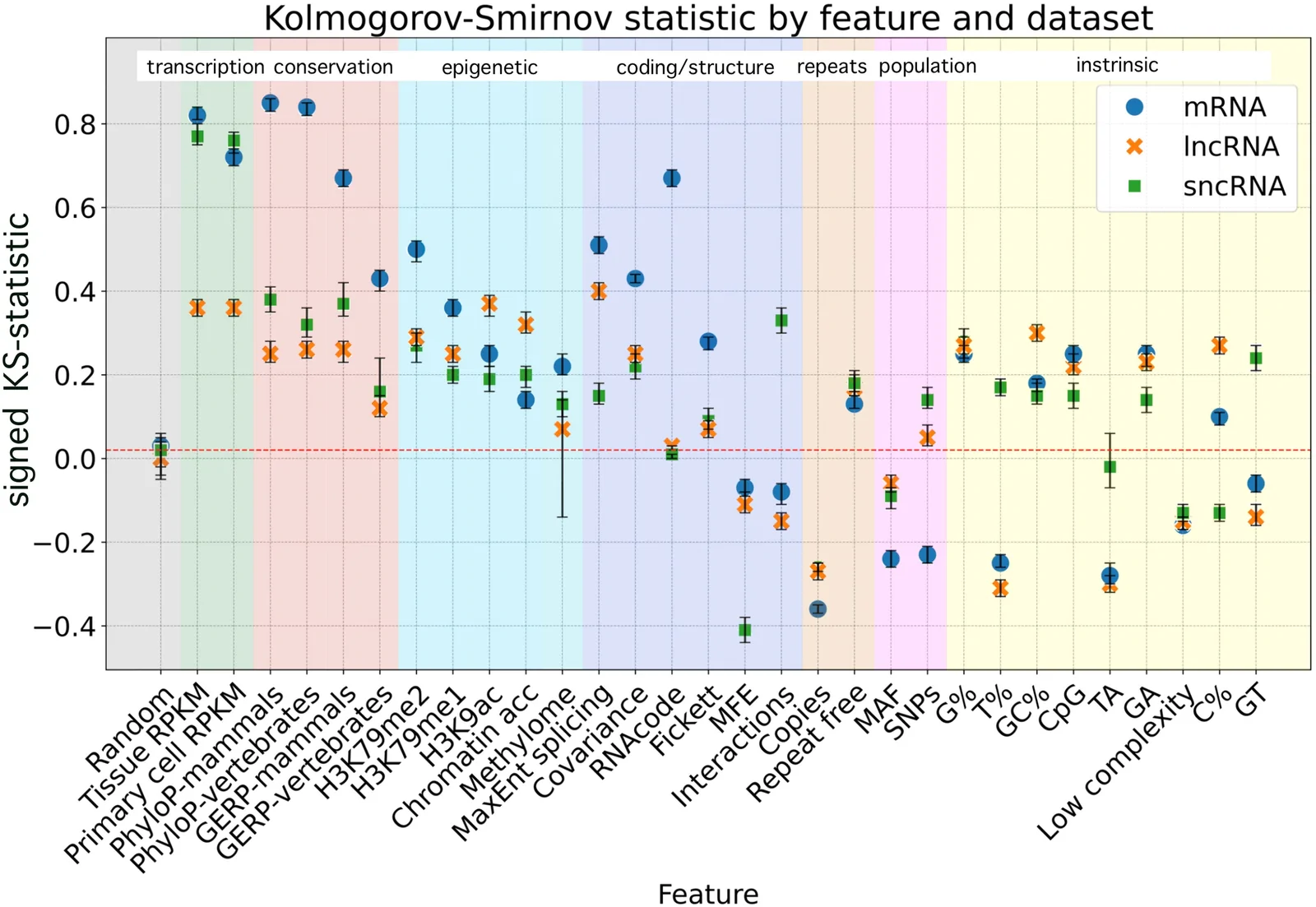
KS statistics by feature and dataset. Signed KS statistics comparing functional sequences to the negative control for each feature and dataset. Each point represents the statistic with the 95% confidence intervals, calculated using bootstrapping (1,000 replicates). The horizontal dashed red line indicates the value for the Random feature, and the background shading the feature type.

### Robust *z*-scores for features

In order to place all genomic features on comparable scales, we computed robust *z*-scores (Rousseeuw and Croux 1993). These also allow comparable assessments of differences between the positive and negative control sequences for each feature. Given the very extreme distributions in our genomic data, we have favored robust *z*-scores which use the median and the median absolute deviation (MAD) compared to more sensitive means and standard distributions. The following formula was used (Rousseeuw and Croux 1993):


$$Z_{i}=\frac{x_{i}\;-\;\mathrm{median}(x_{j})}{1.4826\;\times \;\mathrm{MAD}(x_{j})}\;$$

$$\mathrm{MAD}(x_{i})=\mathrm{median}|x_{i}-\;\mathrm{median}(x_{j})|\;$$

where *x_j_* is the data corresponding to negative control sets for protein coding, short or long ncRNA. That is, each dataset for each gene type is normalized such that the corresponding negative control has a median of zero and a standardized spread. This normalization improves both for visualization—by placing features on comparable scales—and for statistical evaluation across data types. However, despite this normalization, some panels use independent *y* axis scales to accommodate features with highly unequal variance and to allow their distribution to be visualized without excessive compression.

### KS statistics for features

We assessed the direction and absolute difference in distributions between positive and negative control sequences using a signed KS test. We selected this test for its robustness and nonparametric nature, which allowed us to compare distributions without assuming an underlying data distribution (Kini et al. 2025). The KS test's ability to detect location and shape differences in empirical distributions makes it useful for detecting subtle but significant variations. We used the ks.test and p.adjust functions within the standard R pdackage “stats” v 4.4.1 to assess significance, and then *P*-values were adjusted using BH/FDR correction across all features (number tests were equal to the number of features −> 31 in total). To obtain the direction of the stochastic difference between distributions, we computed the cumulative distribution function's maximal and minimal difference and returned the signed value. The confidence intervals were calculated using a bootstrap approach (*N* = 1,000). To interpret the magnitude of the KS statistic, we established the following thresholds: strong (|KS| statistic > 0.4), moderate (0.2 < |KS statistic| ≤ 0.4), and weak (|KS statistic| ≤ 0.2).

## Results

Our study assesses the statistical relevance of 6 classes of genomic features for determining gene functionality. We include sequence conservation, transcription levels, epigenetic signatures, genomic repeat associations, protein and RNA-specific scores, and population variation (Fig. 1). We compare these features across 3 main categories of genes—protein-coding (mRNA), sncRNA, and lncRNA—against length-matched nongenic control regions. The selected genomic features have independently been associated with function in previous analyses and were selected from a range of statistics and scores that met the following inclusion criteria: readily accessible for human genome version GRCh38/hg38, are genome-wide, unbiased to known genes, and not redundant (Supplementary Tables 1 and 2 for further details of included and excluded features; see also Spearman correlation matrices: Supplementary Figs. 4 to 6). Here we present each analysis and discuss the biological relevance to the definition of functionality.

### All features in context

As shown in Fig. 1, the most important features that clearly differentiate the different functional genes types and their corresponding negative controls are expression (tissue and primary-cell RPKM) and evolutionary conservation (phyloP, GERP). These 2 are followed by splicing (MaxEnt splicing), chromatin state (H3K79me2), and repeat association (copies). These features show the strongest effects across all 3 main gene types, protein-coding (mRNA), sncRNA, and lncRNA.

Further discriminators of particular note are that coding potential (RNAcode) and secondary structural signatures (covariance) are key important discriminators for protein-coding genes, while secondary structure (MFE) for sncRNA genes and chromatin state (Chromatin) for lncRNA genes distinguish genic from intergenic regions (Supplementary Fig. 7).

### Transcription

Transcription is an essential step for the expression of both protein-coding and non-coding genes (Vannini and Cramer 2012; Poliseno et al. 2024). While all genes are transcribed, it is a logical fallacy (affirming the consequent) to assume that all transcribed regions are genes, due to the possibility of transcriptional noise (Raser and O'Shea 2005; Palazzo and Lee 2015; Doolittle and Brunet 2017; Doolittle 2018). To assess transcription signals, we use maximum RPKM (reads per kilobase of transcript per million mapped reads) to summarize transcription across ENCODE Total and Small RNA-Seq experiments from 71 human tissues and 140 human primary-cell samples (ENCODE Project Consortium 2012).

The effect size for the differences between positive and negative controls for expression features is high (KS statistics range from 0.36 to 0.82) (Fig. 2; Supplementary Table 5), suggesting that genes are highly transcribed compared to negative control sequences. In addition, the robust *z*-scores contain rather extreme values (*z* ranges from −1.2 to 1.26 M for sncRNAs) (Fig. 2; Supplementary Table 6; Supplementary Fig. 8). This is indicative of extreme differences between the positive and negative sets.

**Fig. 2.**
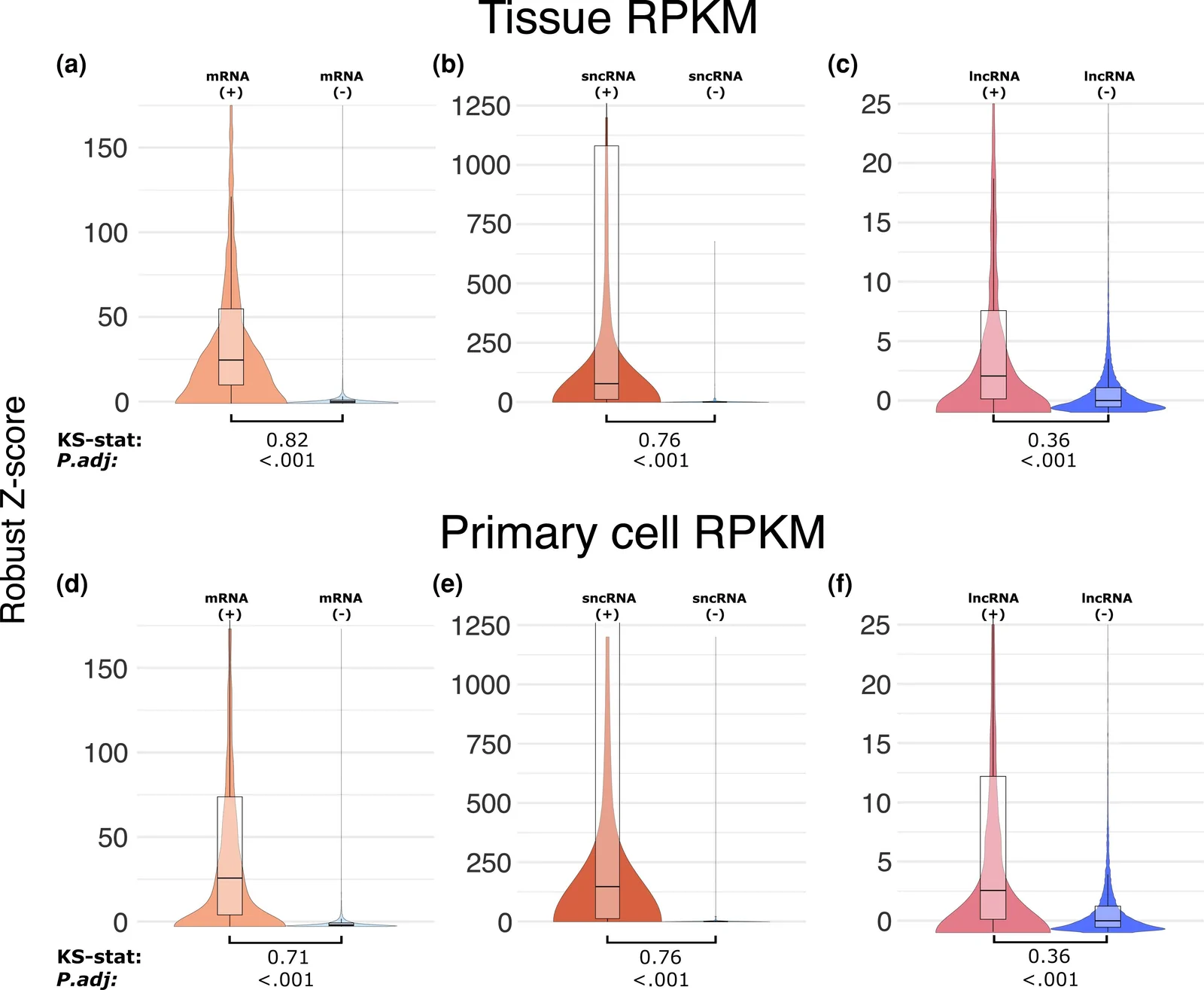
Violin plot and box plots for the distribution of transcriptome data. a to f) Robust *z*-scores for genic and nongenic maximum RPKM across samples (ie the highest normalized expression value from RNA-seq data, summarized as reads per kilobase per million (RPKM) mapped reads). Robust *z*-scores are computed based upon median and median-absolute-deviation values from the nongenic controls. a to c) Correspond to tissue samples. d to f) Correspond to primary-cell samples. Signed KS statistics and adjusted *P*-values comparing genic and nongenic controls are displayed under each pair of positive and negative controls.

### Conservation

Evolutionary conservation between species is an indicator of function, as negative selection purges variation in these regions due to harmful effects on fitness (Cooper et al. 2005; Nielsen et al. 2007; Christmas et al. 2023). In contrast, nonfunctional sequences evolve at the neutral rate, which accumulate 1 to 1.5 mutations per 10 megabases per generation (Bergeron et al. 2023). The genome evolution of species with small effective population sizes, such as humans, is consequently dominated by drift, and therefore fixes many of these mutations (Lynch et al. 2011).

Conservation is assessed here using the phyloP (Pollard et al. 2010) and GERP methods (Cooper et al. 2005; Davydov et al. 2010). We selected datasets that cover both long and short evolutionary distances for each conservation method. For phyloP, we used the short-distance 241-way mammalian dataset (Christmas et al. 2023) and the long-distance 100-way vertebrate dataset from UCSC genome database (Raney et al. 2024). For GERP, we used the short-distance 91-way mammalian dataset and long-distance 63-way amniote vertebrate dataset from Ensembl release 111 (Harrison et al. 2024).

The effect size for differences between positive and negative controls is significant for all conservation features (KS statistics range from 0.12 to 0.84). This is most pronounced for protein-coding genes: phyloP 241-way mammals (KS statistics 0.84) and phyloP 100-way vertebrates (KS statistics 0.83) with somewhat lower effects for the GERP scores. These results indicate high evolutionary conservation for both protein-coding and short non-coding genes (Fig. 3; Supplementary Table 5; Supplementary Fig. 9). The lncRNAs, in contrast, showed limited, but not insignificant levels of evolutionary conservation (KS statistics 0.12 to 0.24), confirming earlier analyses of lncRNA conservation (Kutter et al. 2012; Eddy 2014; Hezroni et al. 2015).

**Fig. 3.**
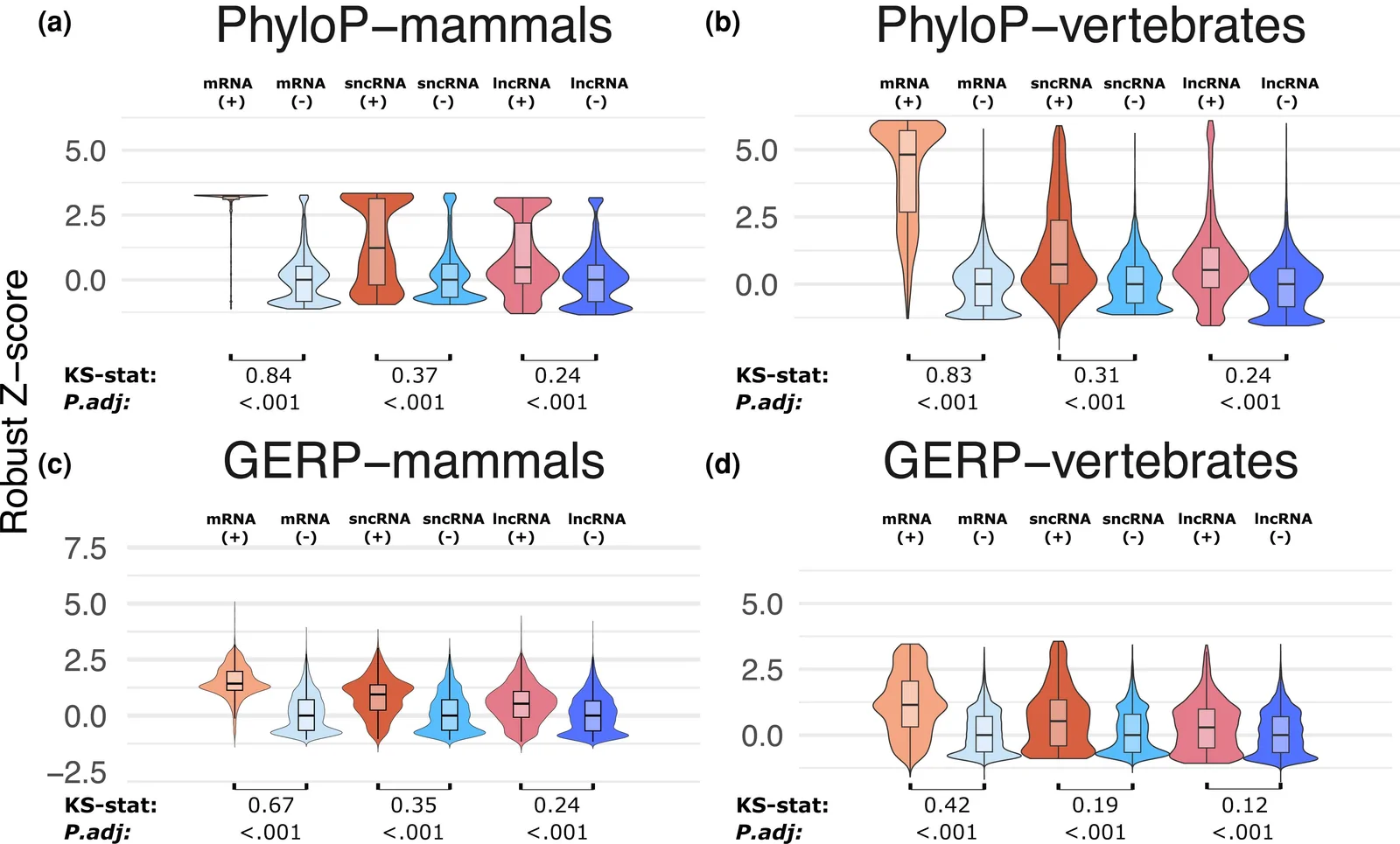
Violin plot and box plots for the distribution of conservation features based on robust *z*-scores for genic and matched nongenic regions. a and b) PhyloP evolutionary conservation scores for the 241-way placental mammal and 100-way vertebrate alignments, respectively. c and d) GERP evolutionary conservation scores for a 91-way eutherian mammal and 63-way amniote vertebrate alignments, respectively. The signed KS statistics and adjusted *P*-values are displayed under each pair of positive and negative controls.

The conservation signatures are generally greater for the “short” evolutionary distance mammalian alignments, which may indicate the difficulties of accurately aligning divergent nucleotide sequences from long evolutionary distances (Gardner et al. 2005). Generally the phyloP scores outperformed GERP for distinguishing genes from nongenic regions.

### Epigenetic features

“Epigenetic” data, such as chromatin accessibility and covalent modifications of DNA or chromatin, may be informative of gene activation or suppression (Bannister and Kouzarides 2011; Allis and Jenuwein 2016; Gershman et al. 2022). Regions of accessible chromatin have been associated with active transcription, while compact chromatin indicates transcriptional repression. Open chromatin regions are usually assayed through nuclease or transposon-based experiments such as DNase-seq and ATAC-seq (Calo and Wysocka 2013; Chawla et al. 2021). In contrast, cytosine methylation is assayed with bisulfite sequencing and has been associated with transcriptional silencing, which may be indicative of nongenic and regulatory regions (Jones 2012; Gelfman et al. 2013; Li et al. 2018; Greenberg and Bourc’his 2019). Histone modifications regulate DNA accessibility and chromatin structure, providing indications of gene regulatory activity across the genome (ENCODE Project Consortium 2012; ENCODE Project Consortium et al. 2020). In this study histones H3K79me2, H3K79me1, and H3K9ac were selected for optimal discrimination between positive and negative control sequences and minimal redundancy with other modification signatures. We have assessed the epigenetic datasets from ENCODE 3 to determine if this information adds further value for identifying canonical genes (ENCODE Project Consortium et al. 2020).

The effect sizes distinguishing the positive and negative controls with epigenetic features are moderate for chromatin accessibility and methylation (KS statistics: 0.12 to 0.32) (Fig. 4; Supplementary Table 5; Supplementary Fig. 10), and comparable to more accessible nucleotide enrichment metrics (eg G%, C%, T%, GC%, CpG, TA, GA content; Supplementary Table 5; Supplementary Fig. 7). However, both chromatin accessibility and methylation are confounded by sequence composition—GC% and CpG, respectively—with Spearman correlations around 0.5, indicating ∼25% of the variance of these genomic datasets is driven by sequence composition (Supplementary Table 7; Supplementary Figs. 4 to 6). Additionally, both chromatin accessibility and methylation features show weak correlations with transcriptional activity (tissue RPKM and primary-cell RPKM; mean Spearman's rho = 0.11 and 0.07) (Supplementary Table 7), consistent with other studies (Kiani et al. 2022).

**Fig. 4.**
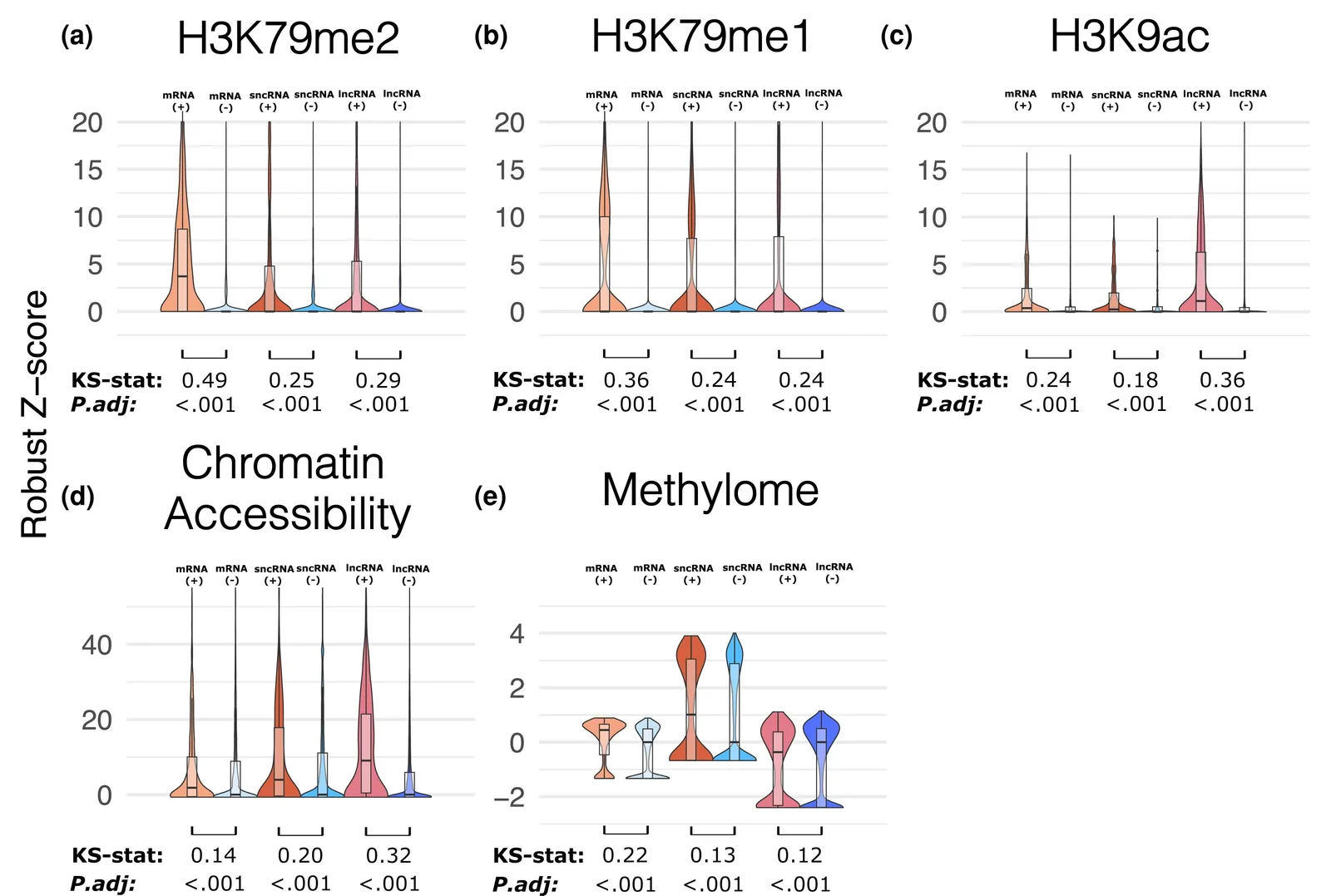
Violin plot and box plots for the distribution of epigenetic features based on robust *z*-scores for genic and matched nongenic regions. a to c) H3K79me2, H3K79me1, and H3K9ac, maximum signal value for these histone marks from ChIP-seq data. d) Chromatin accessibility, the maximum signal value for open chromatin regions from DNase-seq and ATAC-seq data. e) Methylome, the average methylation level at CpG sites from bisulfite-sequencing data. All “epigenetic” data were sourced from the ENCODE3 repository. The signed KS statistics and adjusted *P*-values are displayed under each pair of positive and negative controls.

In contrast, the effect sizes are comparatively higher for the selected histone marks H3K79me1, H3K9ac, and H3K79me2 (KS statistics range from 0.18 to 0.49) (Fig. 4; Supplementary Table 5; Supplementary Fig. 3). Each feature has a weak to moderate correlation with transcription (Spearman's rho ranges from 0.06 to 0.35) (Supplementary Table 7), indicating these may be useful orthogonal measures of functional genes.

### mRNA and ncRNA features: structure, interactions, and coding potential

#### Protein-coding potential features

A number of methods have been developed for differentiating between coding and non-coding sequences, using metrics such as open-reading frame length, codon usage bias, and weighted nucleotide frequencies (Bahiri-Elitzur and Tuller 2021; Champion et al. 2025). While it is unlikely that coding potential can be used to differentiate between ncRNAs and the negative control regions, it is likely that assigned functional protein-coding genes can be classed as “coding,” whereas the negative controls will be “non-coding.”

Two independent and accurate metrics were selected to capture coding potential information based on a recent benchmark (Champion et al. 2025): RNAcode uses MSAs to calculate a coding potential score based on variation patterns (Washietl et al. 2011), whereas the Fickett score uses compositional bias between codon positions from individual sequences (Fickett and Tung 1992).

As expected, the distributions of RNAcode and Fickett scores for the functional protein coding RNA dataset show large differences from the negative controls (KS statistics 0.67 and 0.28, respectively), suggesting that it is likely associated with protein-coding functionality, while the ncRNAs effect sizes were weak (KS statistics ranged from 0.01 to 0.09) (Fig. 5, a and b; Supplementary Table 5).

**Fig. 5.**
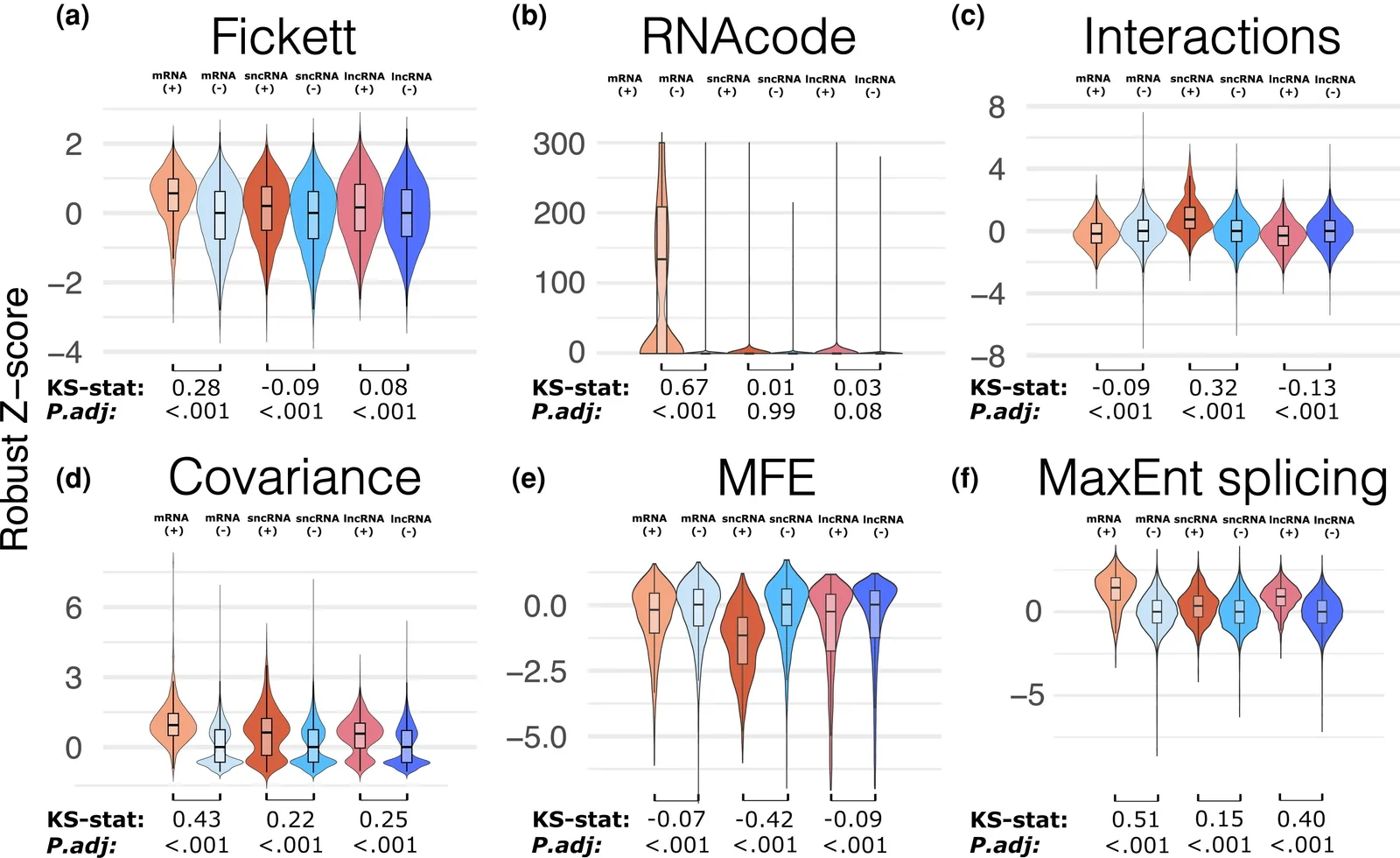
Violin plot and box plots for the distribution of mRNA and ncRNA-specific features based on robust *z*-scores for genic and matched nongenic regions. a and b) Fickett and RNAcode, coding potential based on a measure of nucleotide composition bias and positional asymmetry/or evolutionary signatures of purifying selection on synonymous sites within MSAs, respectively. c) Interactions, average interaction free energies between sequences and 34 abundant human ncRNAs from RNAcentral. d) Covariance, measure of evolutionary pressure to maintain RNA base-pair interactions, reflecting structural constraints. e) MFE, lowest thermodynamic energy of the most stable predicted RNA secondary structure. f) MaxEnt splicing, sum of 5′ and 3′ splice-site scores which capture canonical splice site propensities. Signed KS statistics and adjusted *P*-values are displayed under each pair of positive and negative controls.

#### RNA structure features

Many functional ncRNAs maintain conserved RNA secondary structures that can be detected with pairwise covariation statistics in sequence alignments (Chiu and Kolodziejczak 1991; Kertesz et al. 2010; Li et al. 2012; Ding et al. 2014; Rivas et al. 2017). A nearest-neighbor model of free energy of RNA has been widely used to infer stable secondary structures; however, robust controls are required to correct for C + G content, dinucleotide, and length to control for confounding factors (Workman and Krogh 1999; Rivas and Eddy 2000).

To capture covarying sites in alignment, which can indicate conserved RNA base pairs, we used the maximum *G*-test covariance score implemented in R-scape (Rivas et al. 2017). The effect size for covariance on the difference between functional and negative controls is large for protein (KS statistic 0.43), while the lncRNA and sncRNAs have a more moderate difference (KS statistic 0.25 and 0.22, respectively) (Fig. 5d; Supplementary Table 5). This finding is unexpected because, outside a limited number of regulatory elements, protein-coding transcripts are not generally thought to preserve long-range RNA secondary structure through compensatory substitutions (Katz and Burge 2003; Rivas et al. 2017; Rivas 2020). Similarly, most lncRNAs show little convincing evidence of conserved base pairing across species (Haerty and Ponting 2014; Palazzo and Lee 2015; Ulitsky 2016). The moderate signal observed for short ncRNAs may reflect the prevalence of classes such as miRNAs and C/D box snoRNAs, where function is frequently mediated by short guide or seed regions and protein interactions, yielding limited detectable covariation in alignments (Brennecke et al. 2005; Gardner et al. 2010; Rivas 2020). Note, some summary measures of covariance (minimum and sum of pairwise covariance and presence/absence of significant covariance) were found to be uninformative on a small initial independent inclusion test; these were subsequently excluded from further analyses (Supplementary Table 2).

Additionally, because some ncRNAs form stable RNA secondary structures with a low free energy, we estimate the MFE of optimal structures for each control sequence with RNAfold (Lorenz et al. 2011). We observed a large effect for the short ncRNAs (KS statistic −0.42), while the effect sizes were small for the mRNA and lncRNAs as expected (KS statistics −0.07 and −0.09, respectively) (Fig. 5e; Supplementary Table 5).

#### RNA:RNA interactions features

While RNA–RNA interactions play key regulatory roles, there is also evidence of selection against stochastic interactions, leading to widespread interaction avoidance signals in non-coding RNAs (Umu et al. 2016; Bhandari et al. 2021). To quantify this, we used RNAup (Mückstein et al. 2008) to compute RNA–RNA binding free energies between each sequence and a set of 34 abundant, conserved ncRNAs (listed in Supplementary Table 4).

Short ncRNAs have higher *z*-scores, suggesting that excess stable RNA–RNA interactions are selected against. We further assessed the strongest sncRNA interactions and found that snoRNA–tRNA interactions are the most common (Supplementary Table 4). This implies that these interactions are not driven by sequence homology, and may further support “orphan” snoRNA-directed tRNA modifications (Zhang et al. 2023) (Supplementary Table 4). The interaction excesses or avoidance signals for protein and lncRNA were comparatively weak (KS statistics −0.09 and −0.13, respectively) (Fig. 5c; Supplementary Table 5).

#### Splicing

Spliced RNAs, including mRNAs and lncRNAs, are processed by the spliceosome, which recognizes the 5′ splice site, branch point, and the 3′ splice site to excise introns and ligate exons (Wilkinson et al. 2020; Staněk 2021). Splice-site strength was estimated with MaxEntScan (Yeo and Burge 2004), by scoring each region, including 50 nt of flanking sequence, with the donor and acceptor models and summing the maximum score from each.

The effect size for MaxEnt splicing on the difference between functional and negative controls is large for protein (KS statistic 0.51), while the lncRNA and sncRNAs have moderate and weak differences, respectively (KS statistic 0.40 and 0.15, respectively) (Fig. 5f; Supplementary Table 5).

### Repeat associations

Repeat elements such as TEs, LINEs, SINEs, and endogenous retroviruses drive major variations in genome sizes (Osmanski et al. 2023). The bulk of repeats are inactive, and evolve at approximately the neutral rate of evolution (Arkhipova 2018). Transposon mutagenesis techniques such as tnSeq can aid the identification of genomic regions required for survival, and have been well used in bacterial genomics research (Hutchison et al. 1999; Cain et al. 2020). This work inspired a “repeat-free” screen of the human genome, which successfully identified portions of the genome that resist transposon insertions (Simons et al. 2006). We have modeled the “repeat-free” method by determining the combined distance to the nearest upstream and downstream nonoverlapping repeat annotation. These include TEs, satellite repeats, and pseudogenes from the Dfam database (Hubley et al. 2016; Storer et al. 2021). We excluded repeats overlapping gene annotations, as many functional sncRNA genes are classed as repeats by sequence similarity-based annotation tools (eg tRNA-derived SINES and the SRP RNA-derived Alu repeats) (Kriegs et al. 2007; Sun et al. 2007).

The effect size for the differences between positive and negative controls of the repeat-free regions is weak. The signed *D* statistics from KS test are 0.13, 0.17, and 0.16 for proteins, short ncRNAs, and lncRNAs, respectively (Fig. 6a; Supplementary Table 5).

**Fig. 6.**
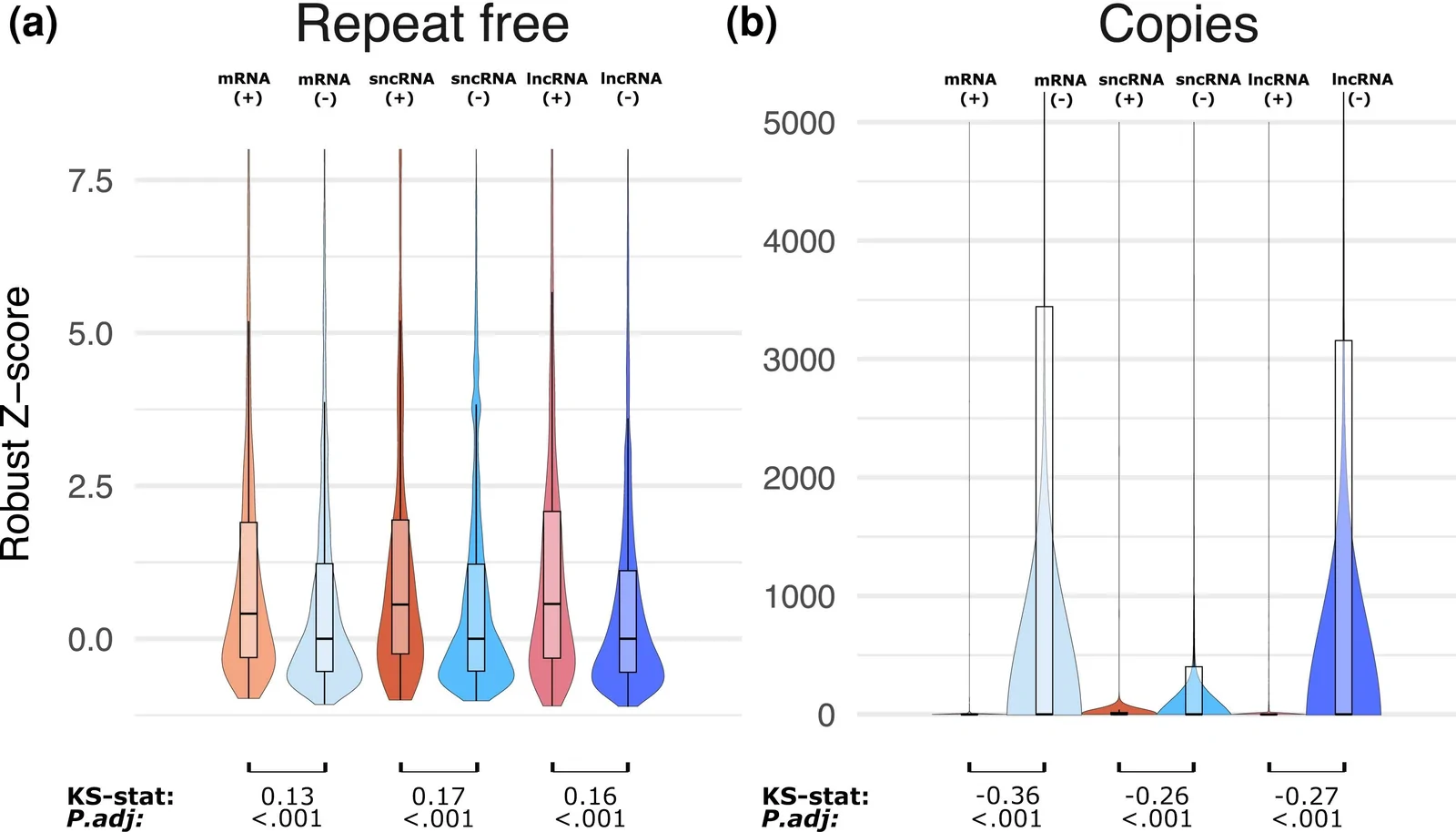
Violin plot and box plots for the distribution of repeat association features based on robust *z*-scores for genic and matched nongenic regions. a) Repeat-free, combined distance to nearest up- and downstream repeat elements (SINE, LINE, etc.) that do not overlap each control sequence. b) Copies is the genomic copy number of each analyzed region as determined by MMseqs2 searches of the GRCh38 genome. Signed KS statistics and adjusted *P*-values are displayed under each pair of positive and negative controls.

We also consider the genomic copy number of each control. Duplicated genes may be more likely to be functional compared to single-copy genes, suggesting that genomic copy number could be a potential feature of function (Ohno 1970; Bergthorsson et al. 2007; Mihajlovic et al. 2025). Alternatively, regions that have very high copy number may indicate pseudogenized transposable elements (Osmanski et al. 2023). Either way, copy number is worth considering as a potential indicator of function.

The copy number results show markedly different distributions between the positive and negative control datasets. Again, the *P*-values are near zero, and the signed *D* statistics from KS test are −0.36, −0.26, and −0.27 for proteins, short ncRNAs, and lncRNAs, respectively. This indicates that genes have far fewer genomic copies than negative control datasets, which have high numbers of repeats (Fig. 6b; Supplementary Table 5; Supplementary Figs. 11 and 12).

Measures based on sequence conservation (phyloP, GERP, RNAcode, covariation) show strong correlations with genomic copy number (Spearman's rho = 0.42 to 0.62; Supplementary Table 7). While this may partly reflect confounding by sequence composition or complexity, the correlations of these potential confounders with copy number is weak to moderate (Spearman's rho = 0.01 to 0.29; Supplementary Table 7).

### Population variation

Genetic variation between individuals is shaped by selection on function: functionally important regions tend to carry less variation due to purifying selection, while nonfunctional regions accumulate variants more freely (Vitti et al. 2013). To assess this, we use data from gnomAD v4 (Chen et al. 2024) to calculate SNP density and the average MAF for each region.

The effect size for the differences between positive and negative controls with population variation features is weak to moderate for unsigned KS statistics (0.05 to 0.24) (Fig. 7, a and b; Supplementary Table 5). The results suggest that, in general, protein-coding sequences have fewer SNPs (KS statistic −0.23 for SNP density) compared to the negative controls. However, for some short ncRNAs, there are remarkably higher numbers of SNPs (KS statistic 0.14 for SNP density) (Fig. 7, a and c; Supplementary Table 5). We explored the enrichment of SNPs in short ncRNAs further and discovered that a few families of ncRNA were affected by elevated SNP counts: spliceosomal RNAs (snRNA) (0.42 to 2.74 SNPs/nuc), snaR (0.43 to 2.39 SNPs/nuc), tRNAs (0.06 to 2.32 SNPs/nuc), and vault RNA (0.34 to 0.92 SNPs/nuc). Note that in the absence of INDELs, 3.0 SNPs/nuc is the theoretical maximum (Fig. 7c, Supplementary Table 8).

**Fig. 7.**
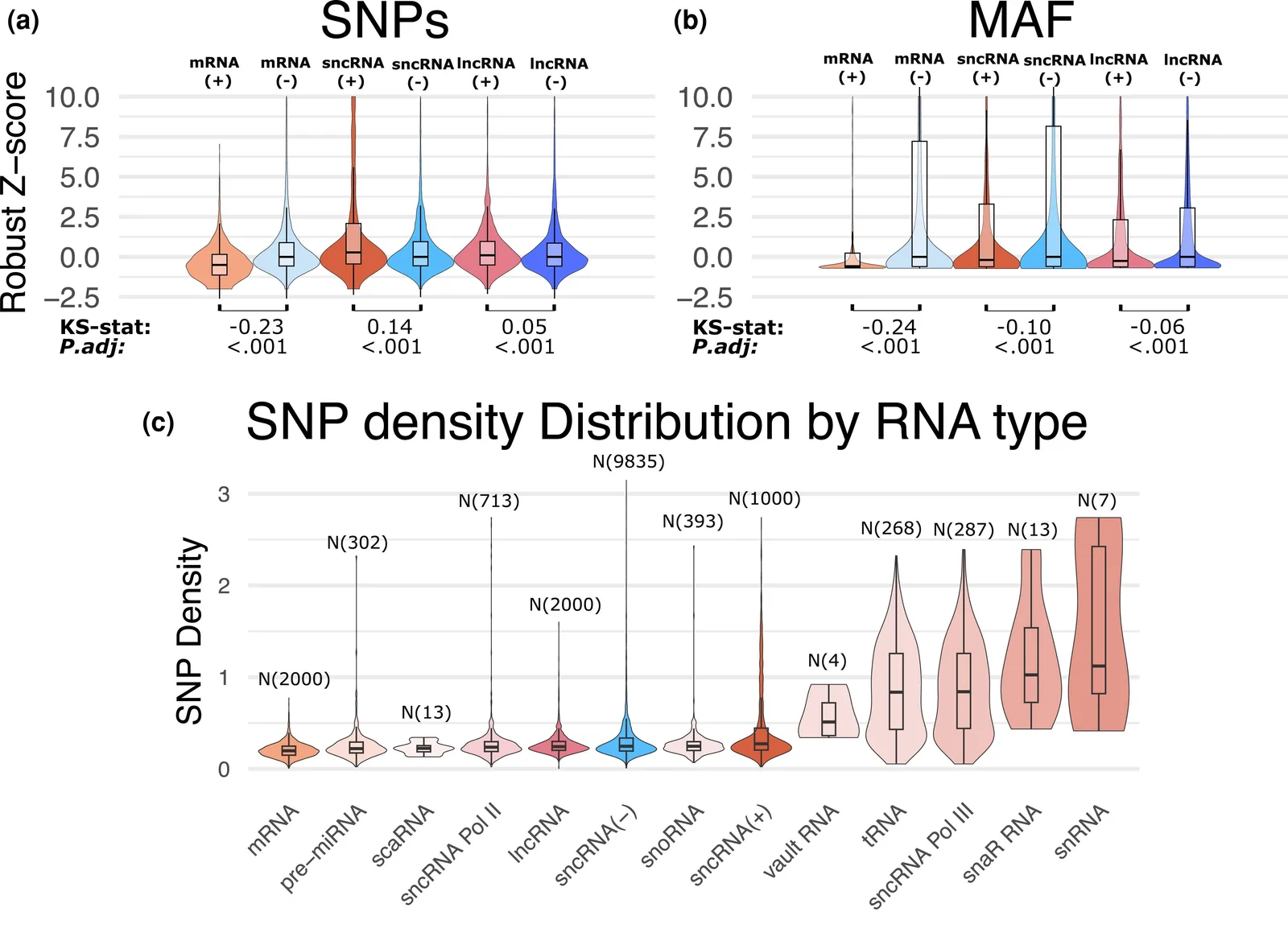
Violin plot and box plots for the distribution of population variation features based on robust *z*-scores for genic and matched nongenic regions. a) SNP density, measured by the number of SNPs/sequence length. b) MAF is the mean minor allele frequency across all SNPs in a region, where the MAF is the population frequency of the least common allele at each variant site. Signed KS statistics and corresponding adjusted *P*-values are displayed under each pair of positive and negative controls. c) The SNP density distributions for different RNA types or polymerase sorted by median SNP density.

Due to the possibility of linkage disequilibrium influencing the SNP statistics, we checked for an association between the distances of each negative control from the corresponding positive control (gene). We found the relationships between distance and SNP statistics (eg density) to be minimal (Spearman's rho of −0.01, −0.03, and −0.01 for mRNA, sncRNA, and lncRNA genes, respectively) (Supplementary Fig. 13; Supplementary Table 9).

SNP density is associated with CpG, low complexity, GC%, and copy number, with mean absolute Spearman's rho values of 0.37, 0.33, 0.28, and 0.23, respectively (Supplementary Figs. 4 to 6; Supplementary Table 7). Many of these genomic features may be associated with an increased mutability and misannotation due to methylations, polymerase slippage, segmental duplicates, and even RNA polymerase type (Cooper and Krawczak 1990; Meunier and Duret 2004; Li 2014; Seplyarskiy et al. 2023).

### Intrinsic sequence features

Intrinsic sequence features are the local components of a sequence. In this work, we include commonly used metrics such as C + G content, the proportions of mononucleotide and dinucleotide frequencies, and the presence of low complexity sequences, which capture simple repeats.

#### Nucleotide statistics

C + G-rich regions have been linked to both protein-coding and non-coding RNA genes (Lander et al. 2001; Haerty and Ponting 2015; Christmas et al. 2025), while internal coding exons show elevated G and C content relative to A and T, with A more abundant than T (Louie et al. 2003).

For the 16 possible DNA dinucleotides, we compare the observed frequency of each dinucleotide to the expected frequencies based on the corresponding mononucleotide frequencies. This should capture CpG sites underrepresentation due to methylation and subsequent TpG conversion (Medvedeva et al. 2010), and the known TA underrepresented in genes, possibly driven by the suppression of cross-talk with cellular processes such as splice site recognition, AU-rich RNA degradation motifs, “TATA” motif, and premature stop codons (Karlin and Mrázek 1997; Barreau et al. 2005; Akinyi and Frilander 2021; Olthof et al. 2024), although a recent paper suggests a role in RNA degradation (Su et al. 2024). Likewise, GT and AG dinucleotides may be avoided due to their roles in splice site identification (Olthof et al. 2024). Further differences in dinucleotide composition may be driven by codon bias or RNA folding and interaction requirements (Umu et al. 2016; Parvathy et al. 2022).

LCRs, which consist of repetitive or biased subsequences, deviate from random sequence norms (Enright et al. 2023). Within coding sequences, LCRs are thought to evolve neutrally due to low conservation and size variation, driven by polymerase slippage and unequal recombination. As nonfunctional regions, with potentially detrimental effects, LCRs may be selected against in most genic sequences (Lenz et al. 2014; Enright et al. 2023).

The unsigned *D* statistics for mononucleotide frequencies range from 0.09 to 0.30. Relative to controls, genes show increased G% and C% and decreased T%, with the exception of sncRNA genes, which show the reverse for C% or T%. The signed *D* statistics for the GC% are weak to moderate (KS statistics range from 0.15 to 0.30), supporting the previous reports for coding and non-coding genes (Lander et al. 2001; Haerty and Ponting 2015). Meanwhile, the signed *D* statistics for low complexity density results are lower (−0.12 to −0.16), indicating that the coding and non-coding genic sequences have a higher complexity (fewer repetitive sequences) compared to negative control regions, as expected. For the dinucleotide compositions, the unsigned *D* statistics range from 0.03 to 0.30. The most marked differences can be observed for CpG (increased), GA (increased), and TA (decreased), indicating significant shifts of dinucleotides in positive controls compared to the negative control dataset across all 3 gene types. In contrast, the dinucleotide GT is enriched in the sncRNAs. This is notable as GT is not associated with any known snoRNA or tRNA motif (Fig. 8; Supplementary Table 5).

**Fig. 8.**
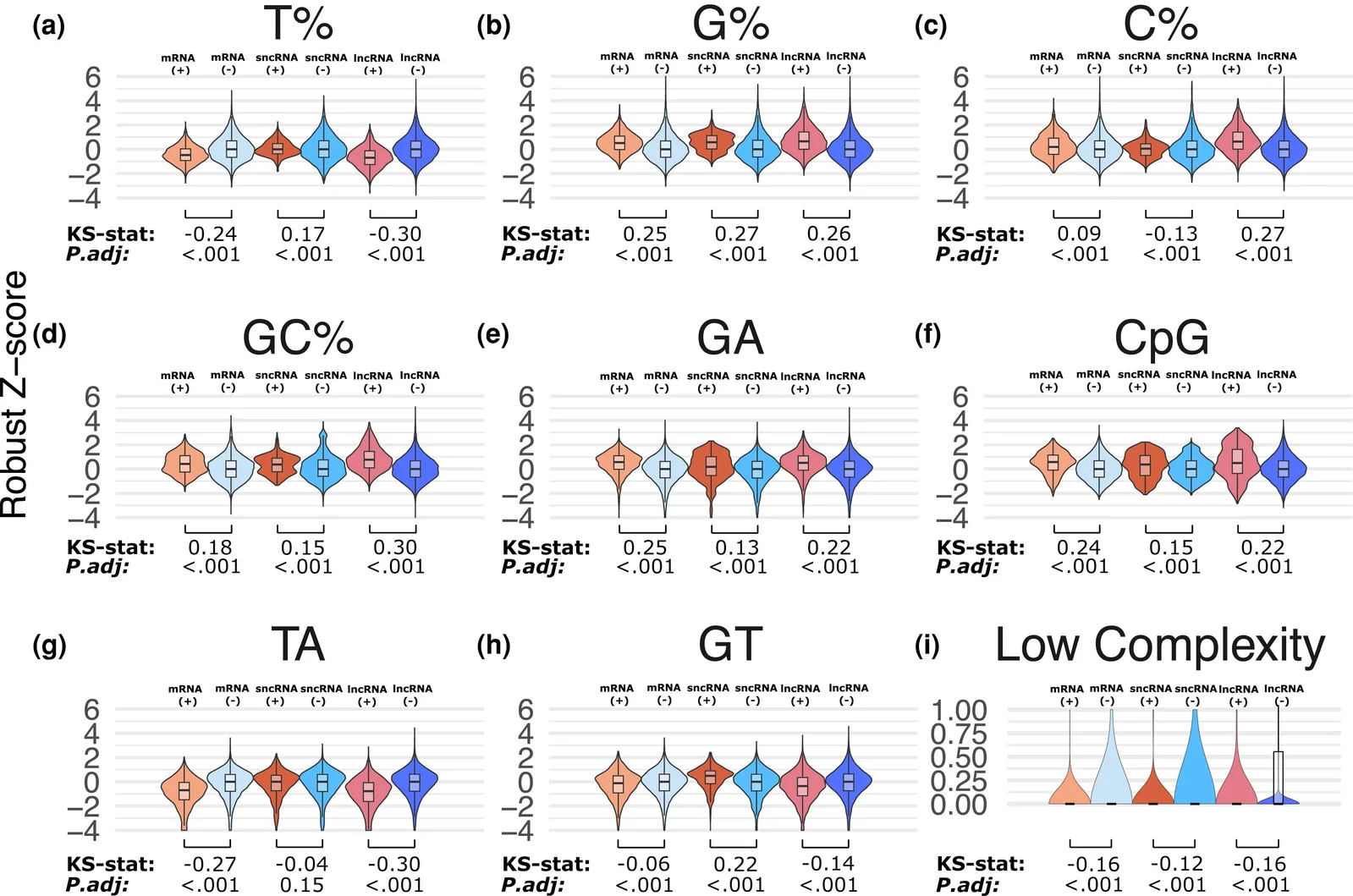
Violin plot and box plots for the distribution of intrinsic sequence features based on robust *z*-scores for genic and matched nongenic regions. a to c) Mononucleotide frequencies for T, G, and C. d) GC%, the C + G proportion for each sequence. e to h) Selected log-odds of dinucleotide proportions relative to mononucleotide frequencies for GA, CpG, TA, and GT, respectively. i) Low complexity, the proportion of sequence not classed as “low complexity” by the DUST annotation tool. The signed KS statistics and adjusted *P*-values are displayed under each pair of positive and negative controls.

## Discussion

In genetics, “function” is interpreted in 2 main ways. The causal role view considers any biochemical activity—like transcription—as functional. In contrast, the selected effect view requires both biochemical activity and evidence of evolutionary selection, acknowledging that some cellular activity may be mere noise (Eddy 2012; Doolittle and Brunet 2017; Graur 2017). Our analysis is based on the premise that genic exons are distinguishable from intergenic regions with genome-wide signals. By examining a broad range of features, we systematically assessed the relative power of different indicators for separating the genic from nongenic regions. Transcriptional activity (RPKM) emerged as the strongest predictor—especially for protein-coding and short ncRNAs, while evolutionary conservation was the next most informative, particularly for coding regions. Conservation, however, was generally a weaker signal for ncRNAs (Figs. 1 to 3; Supplementary Fig. 7).

Together, these results show that combining biochemical activity and evolutionary selection offers a robust framework for identifying functional genomic regions. In addition, our feature-based analysis strongly supports the “selected effect” model of function, where both signal and selection are needed to reliably distinguish genuine genes from genomic noise (Fig. 1; Supplementary Table 5).

Three histone modifications, H3K79me1, H3K79me2, and H3K9ac, showed discrimination between gene and control sets comparable to that of transcriptional and conservation signals. However, their strong correlation with transcription and conservation raises questions about whether they offer additional, independent information. In contrast, methylation and chromatin accessibility data showed limited ability to distinguish functional regions. Given this, annotation efforts might be better focused on more informative (and confounded) features such as GC% and CpG enrichment (Supplementary Fig. 7).

Population variation data, such as SNP density, performed poorly in distinguishing functional from nonfunctional regions. For protein-coding genes, their performance was comparable to basic sequence features like GC% and CpG content, which are useful but far from definitive. Strikingly, some short ncRNA classes exhibited extremely high SNP densities, in some cases nearing saturation level (∼3 SNPs per site; Fig. 7c). The presence of such excess variation in otherwise conserved genes is unexpected and warrants further investigation to identify the biological or technical causes. It has recently been proposed that polymerase III transcription is mutagenic (Seplyarskiy et al. 2023). To investigate this further, we compared the SNP densities of pol II and pol III sncRNA transcripts (Fig. 7c) as well as different classes of sncRNAs. The classes with the highest SNP densities are the spliceosomal sRNAs, snaR RNAs, and tRNAs—each of which is transcribed by polymerase III. The polymerase III transcripts overall have far higher SNP densities than the polymerase II transcripts (signed KS = 0.71, *P-*value < 0.001) as well as the negative control set (signed KS = 0.62, *P-*value < 0.001); additionally, polymerase II transcripts have densities more comparable to the negative control set (signed KS = −0.11, *P-*value < 0.001) (Fig. 7c). Some of these genes may also be shaped by transcription-associated mutational biases, particularly near transcription start sites (Cortés Guzmán et al. 2025; Qiu et al. 2025), or by duplication events not captured in the reference genome (Arthur et al. 2015).

Among alignment-based features, R-scape's covariation score (max. *G*-test) was remarkably effective at identifying functional genes, including protein-coding sequences, despite being developed to detect conserved RNA secondary structure (Rivas et al. 2017; Rivas 2020). This unexpected signal may reflect compensatory variation at synonymous sites, a byproduct of the genetic code itself. Similarly, RNAcode's coding potential metric showed strong predictive power for distinguishing coding regions from background based on sequence alignments. MaxEnt splicing was the strongest feature for (spliced) lncRNA detection, and also strong for mRNA detection. In contrast, the free energy-based features (eg MFE, RNA–RNA interaction scores) were strongest in short ncRNAs, but provided little signal in lncRNAs or protein-coding genes (Supplementary Table 5).

In summary, protein-coding regions are clearly distinguishable from genomic background, supported by strong signals across multiple genomic features including conservation, transcription, coding potential, splicing, histone marks, and covariance (Supplementary Table 5). sncRNAs also show strong functional signatures from transcription and folding MFE, and moderate signals from conservation, mono- and dinucleotide frequencies, copies, and covariance (Supplementary Table 5). In contrast, lncRNAs have strong signals from just splicing and moderate signals from transcription, histone marks, chromatin accessibility, GC%, mono- and dinucleotide frequencies, genomic copy number, covariance, and conservation (Supplementary Table 5). This highlights the need for stringent criteria or improved annotations for lncRNA function, which have historically required remarkably little evidence (eg >1 RNA-seq read) (Xu et al. 2017). Note that the lncRNAs are a highly heterogeneous set of transcripts; our class-level summaries should not be interpreted as applying uniformly to all; the specific cases should require careful, gene-by-gene evaluation.

### Limitations of the study

#### Annotation uncertainty

Some positive control annotations may reflect misannotation or overinterpretation of limited evidence. For example, nearly a third of protein-coding genes in the original human genome draft have since been merged or removed (Hatje et al. 2019; Amaral et al. 2023). Similar issues likely affect non-coding annotations, where pseudogenes or spurious transcription may have been overenthusiastically mislabeled as functional (Prasetyo and Gardner 2024). Unfortunately, there are fewer opportunities for systematic re-curation of non-coding annotations compared to coding ones. Conversely, some negative controls may include unannotated functional elements, although both types of errors are expected to be rare given the decades of intensive curation and discovery efforts in annotating the human genome.

We note that further stratification of the annotated lncRNA set by independent evidence of functionality (eg EVlncRNAs; Zhou et al. 2024) is currently being evaluated and will be presented in a subsequent study.

#### Transcriptome coverage and circularity

While we observe strong associations between gene expression, chromatin marks, and annotated genes, our RNA-seq datasets—though extensive (211 samples)—may miss tissue-specific or condition-specific transcripts. As a result, “unexpressed” regions may not truly be inactive. The discovery of many lncRNAs using RNA-seq and chromatin data (Xu et al. 2017) also introduces potential circularity, inflating associations between those features and lncRNA annotations.

#### Negative control design

Our choice of negative control sequences, intergenic regions matched by location and sequence properties, may not be ideal. Alternative approaches, such as synthetic random sequences or shuffled chromosomes, are rarely available and not suitable for comparing evolutionary or population variation signals (Eddy 2013; Camellato et al. 2024). We also assume that nonfunctional regions of the genome share similar evolutionary and biochemical properties. However, transcription can influence both mutation rate and mutation spectrum (Qiu et al. 2025), influencing intrinsic sequence features independently of selection on function. As a result, inactivated repeat elements such as LINEs, SINEs, and endogenous retroviruses may exhibit similarly heterogeneous signatures if investigated in similar levels of detail.

#### Confounding variables

Some confounding factors may have been overlooked. We addressed the most obvious by matching for length and local GC/CpG content, but deeper latent biases may still affect certain features.

#### Class imbalances

To reflect real genome composition, we deliberately included more negative than positive controls, consistent with prior estimates of functional genome fractions (Galeota-Sprung et al. 2020; Christmas et al. 2023). However, within positive sets, imbalance between RNA classes posed a challenge: sncRNAs are dominated by miRNAs (*n* = 302, 30.2%), snoRNAs (*n* = 393, 39.3%), and tRNAs (*n* = 268, 26.8%), requiring downsampling to avoid overrepresentation in downstream analyses.

## Data Availability

All custom scripts, information on downloaded primary data, and the generated datasets are available on GitHub:0.30: https://github.com/danischiavi/gene-functionality.
